# Response to the letter to the editor entitled “Glucodependence in Cardiorenometabolic Disease: Naming the Problem”

**DOI:** 10.1016/j.ekir.2026.106706

**Published:** 2026-07-18

**Authors:** Luxcia Kugathasan, Marcel H.A. Muskiet, Vikas S. Sridhar, David Z.I. Cherney

**Affiliations:** 1 Division of Nephrology, Department of Medicine, University Health Network, Toronto, Ontario, Canada; 2 Division of Endocrinology, Department of Internal Medicine, Leiden University Medical Center, Leiden, The Netherlands; 3 Division of Nephrology, Department of Medicine, University of British Columbia, Vancouver, British Columbia, Canada

The Author Replies:

We thank Dr. Lopez Giacoman for their thoughtful letter^1^ regarding our review, *Novel Therapeutic Strategies for Patients With CKD and Diabetes Mellitus*,^2^ and for highlighting the role of compulsive eating behaviors and the modern food environment in cardio-kidney-metabolic disease. The proposed concept of “glucodependence” offers a thought-provoking framework for maladaptive consumption of sugar-rich and ultraprocessed foods and broadens the discussion by emphasizing behavioral and environmental determinants of long-term outcomes.

Our review focused on emerging pharmacologic therapies with demonstrated or potential cardiorenal benefits in chronic kidney disease and diabetes.^2^ However, we also recognize that lifestyle and dietary behaviors remain central to disease management, and that sustaining healthy food choices is a key determinant of long-term outcomes. As glucagon-like peptide-1-based therapies evolve, understanding their interaction with eating behavior, food choices, and the broader food environment is an important area for future research.

Food-related behaviors are increasingly viewed as complex neurobehavioral processes shaped by reward pathways, environmental exposures, socioeconomic factors, learned behaviors, and widespread access to highly palatable ultraprocessed foods.^3^ These mechanisms may manifest as persistent food-related thoughts (“food noise”), cue responsiveness, cravings, and compulsive eating that complicate sustained dietary change. In a controlled inpatient feeding study, ultraprocessed diets led to greater energy intake and weight gain than minimally processed, nutrient-matched diets, highlighting the role of processing and palatability.^4^ Together, these factors may limit adherence to lifestyle recommendations and contribute to residual cardio-kidney-metabolic risk despite optimal therapy.

Emerging evidence suggests that glucagon-like peptide-1-based therapies influence appetite, satiety, food preference, and central reward signaling, raising the possibility that some of their clinical benefits extend beyond glycemic control and weight reduction.^5^ Early preclinical and clinical evidence also suggests that glucagon-like peptide-1-based therapies may modulate broader reward-related behaviors, including alcohol and/or tobacco use and other addictive behaviors, although these observations require confirmation in dedicated clinical trials.^6^ Whether “glucodependence” represents a distinct, clinically relevant construct remains uncertain. Further research is warranted to better understand the interplay between glucagon-like peptide-1-based therapies, eating behavior, and the food environment. Nevertheless, optimizing long-term cardio-kidney-metabolic outcomes in patients with chronic kidney disease and diabetes will likely require integration of pharmacological approaches with behavioral interventions and strategies that address the food environment.
